# Prognostic significance of hepatocyte growth factor activator inhibitor type 1 (HAI-1) immunoreactivity in pancreatic ductal adenocarcinoma

**DOI:** 10.1186/s13104-017-3014-x

**Published:** 2017-12-04

**Authors:** Chihiro Sakugawa, Yukihiro Haruyama, Hiroyuki Tanaka, Tsuyoshi Fukushima, Makiko Kawaguchi, Hiroaki Kataoka

**Affiliations:** Section of Oncopathology and Regenerative Biology, Department of Pathology, Faculty of Medicine, University of Miyazaki, 5200 Kihara, Kiyotake, Miyazaki, Japan

**Keywords:** HAI-1, SPINT1, Pancreatic ductal adenocarcinoma, Immunohistochemistry

## Abstract

**Objective:**

Hepatocyte growth factor activator inhibitor type 1 (HAI-1) is a membrane-bound serine protease inhibitor that is expressed on the surface of epithelial cells. Evidence has suggested that decreased cell surface HAI-1 in carcinoma cells results in enhanced invasiveness. However, little is known regarding the expression of HAI-1 in pancreatic ductal adenocarcinoma (PDAC). This study aimed to analyze HAI-1 expression in PDAC and its impact on patient prognosis.

**Results:**

HAI-1 immunohistochemistry was performed on samples from 67 PDAC cases. HAI-1 expression was increased in intraepithelial neoplasia compared to the adjacent non-neoplastic ductal epithelium. Of the 67 samples tested, 58% (39/67) of PDAC cases showed diffuse (> 75%) immunoreactivity in PDAC cells. The remaining cases showed reduced HAI-1 immunoreactivity in a substantial number of cancer cells. Although there was no correlation between HAI-1 status and tumor size, histologic grade or lymph node metastasis, diffuse HAI-1 positive cases showed longer disease-free survival (DFS; *p* = 0.006, log-rank test). In conclusion, HAI-1 is upregulated in pancreatic intraepithelial neoplasia and broadly expressed in PDAC cells. However, PDAC cases having areas of reduced HAI-1 immunoreactivity may show shorter DFS.

**Electronic supplementary material:**

The online version of this article (10.1186/s13104-017-3014-x) contains supplementary material, which is available to authorized users.

## Introduction

Pancreatic ductal adenocarcinoma (PDAC) remains a challenging disease with a 5-year survival rate of < 5%. The lethal nature of this disease is attributed largely to significant invasiveness and rapid metastatic spreading [1]. The cancer cell microenvironment plays a critical role in malignant progression and consists of a complex mixture of neoplastic cells and host-derived stromal cells, such as immune/inflammatory cells, cancer-associated fibroblasts (CAF), and endothelial cells. There are also dynamic modifications to the extracellular matrix and bioactive proteins expressed on and secreted from cancer cells and stromal cells, in which pericellular proteolysis plays a significant role [2, 3]. The mutual interactions between cancer cells and stromal cells may be particularly important in PDAC, as it is well known that PDAC tissues usually have abundant CAF [4]. One typical example of this mutual interaction is hepatocyte growth factor (HGF). Cancer cells stimulate stromal fibroblasts to secrete inactive HGF proform (proHGF). After activation by an extracellular protease, mature HGF transduce signals through MET receptor tyrosine kinase and induce pleiotropic effects on cancer cells, such as epithelial to mesenchymal transition (EMT), invasiveness, survival and growth [5].

HGF activator inhibitor type 1 (HAI-1), encoded by the *SPINT1* gene, is a type 1 transmembrane serine protease inhibitor expressed on the surface of most epithelial cells [6, 7]. HAI-1 inhibits major proHGF-activating proteases, such as HGF activator (HGFA), matriptase, hepsin [8, 9]. These proteases are reported to be increased in cancer tissue and contribute to cancer progression [3]. Insufficient HAI-1 levels may result in deregulated pericellular activities of these proteases and accelerate cancer progression. Indeed, loss of cell surface HAI-1 frequently occurs in cancer cells in vivo due to decreased *SPINT1* mRNA levels and/or enhanced shedding of the HAI-1 extracellular domain [10, 11]. We previously reported that S2-CP8, a metastatic subline of the SUIT-2 pancreatic adenocarcinoma cell line, showed markedly decreased HAI-1 expression that accompanied an EMT phenotype [12]. Consequently, HAI-1 knockdown induced EMT of SUIT-2 cells, which was accompanied by enhanced metastatic spreading [13, 14]. However, little is known about HAI-1 expression in PDAC, and its precursor lesion in vivo.

In this study, we performed an immunohistochemical analysis of HAI-1 expression in PDAC using surgically resected PDAC tissues. We also evaluate the impact of HAI-1 expression on patient prognosis.

## Main text

### Methods

The study protocol was in accordance with the revised Helsinki Declaration of 1983 and approved by the Institutional Review Board of the Faculty of Medicine, University of Miyazaki. We reviewed records of PDAC patients who had undergone surgical resection between 2004 and 2010 at the University of Miyazaki Hospital. A total of 67 Japanese patients (34 males and 33 females; between 43 and 85-years-old with a median age of 70) fulfilled the inclusion criteria: (i) definitive pathological diagnosis of PDAC; (ii) availability of formalin-fixed paraffin embedded tissue sections; and (iii) complete clinicopathologic and follow-up data. Exclusion criteria were (i) participants who refused to be included in this study; (ii) cases of recurrent tumor or multiple cancers. T-stage was determined by the definitions provided by the American Joint Committee on cancer (7th edition) [15]. Post-operative overall survival (OS) and disease-free survival (DFS) were defined as the time from the date of surgery to the date of death and the date of initial detection of local PDAC recurrence or distant metastasis, respectively. Postoperative follow-up included abdominal ultrasonography or computed tomography study every 3 months and laboratory testing of serum carcinoembryonic antigen, CA19-9, DUPAN2, s-pancreas-1 antigen levels at 1- to 2-month intervals. Detail has been removed from these case descriptions to ensure anonymity.

To detect HAI-1 expression, 10% formalin-fixed, paraffin embedded tissue sections with a thickness of 5 μm were subjected to immunohistochemical staining. The staining was carried out on a Ventana Discovery automated immunostainer (Roche Diagnostics, Tokyo, Japan) according to the manufacturer’s instructions with a 30 min heat treatment for antigen retrieval. One representative tissue section of a main tumor portion and a section consisting of both tumor and non-tumor pancreatic tissue were immunostained for each case. The primary antibody used was anti-HAI-1 mouse monoclonal antibody (clone 1N7, 10 μg/ml) [7]. The primary antibody was omitted for negative controls. In selected cases that showed weak and focal HAI-1 immunoreactivity, additional negative controls were prepared using non-specific mouse IgG instead of primary antibody. After immunodetection, tissue sections were counterstained with Mayer’s hematoxylin. Most sections included surrounding non-neoplastic pancreatic tissue, and ductal epithelial cells at the site of acinar–ductal metaplasia (ADM) were used as for internal positive controls for HAI-1.

To evaluate HAI-1 expression levels in PDAC cells, we judged HAI-1 immunoreactivity that was similar to or stronger than that of ductal epithelial cells of ADM as positive. Immunoreactivity was then graded using a scale from 1 to 3 according to the ratio of the positive area in cancer tissue as follows: 1, positive in < 50% of cancer cells; 2, ≥ 50 and < 75%; 3, ≥ 75%. Chi square test was used for assessment of associations between variables. OS and DFS were estimated using the Kaplan–Meier method where groups were compared using the log-rank test. Cox proportional hazards regression models were used to calculate the hazard ratios (HRs) and 95% confidence intervals (CIs). Patients were censored on the date of last contact or death due to causes other than PDAC. The multivariable Cox proportional hazards regression analysis model was used to detect independent prognostic factors. Statistical significance was assumed with *p* < 0.05. Data were analyzed by STAT view 5.0 (SAS Institute, Cary, NC).

### Results

**Fig. 1:**
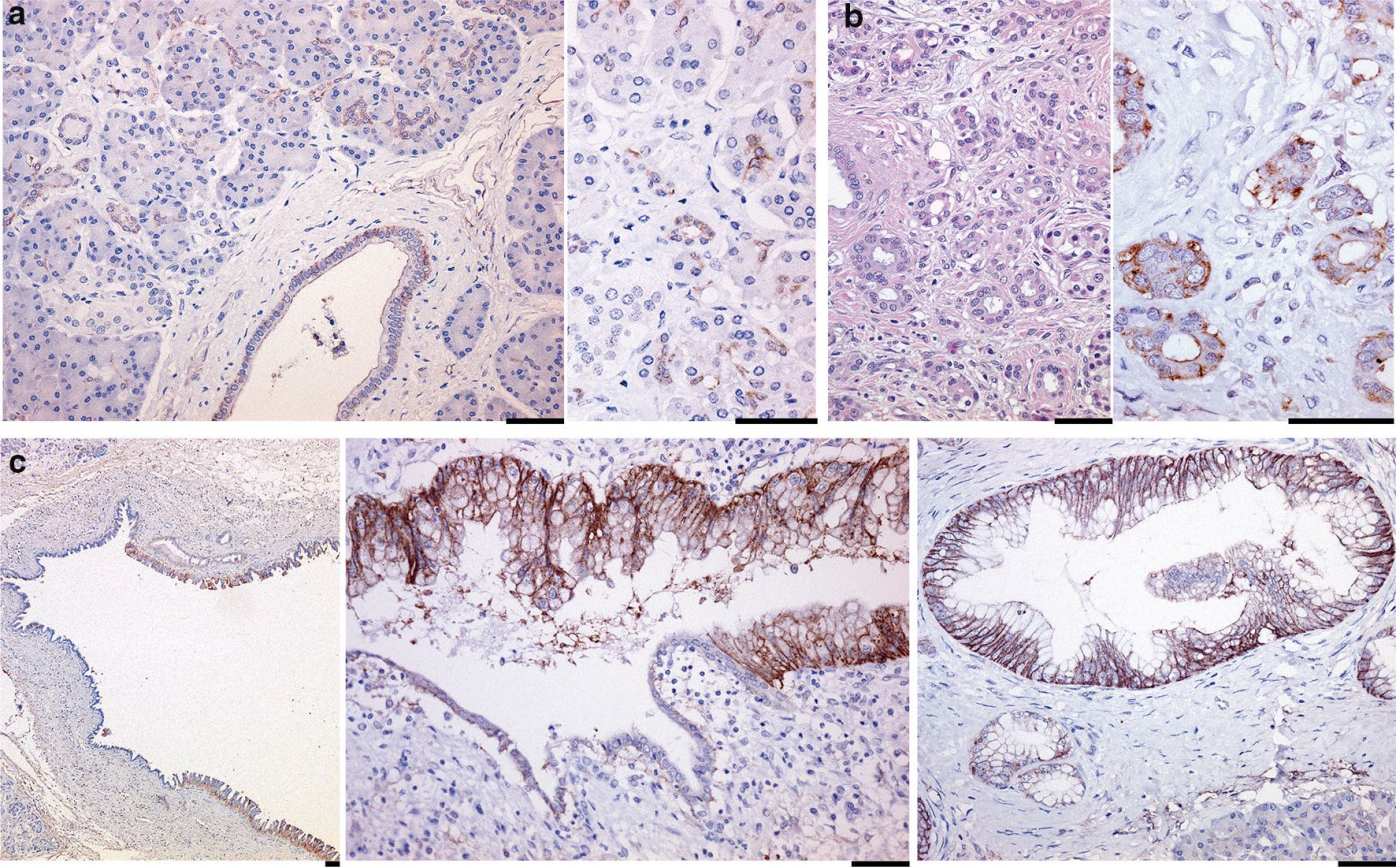
Immunoreactivity of HAI-1 in non-neoplastic pancreatic tissue, ADM and intraductal lesion. **a** HAI-1 immunoreactivity in normal pancreatic tissue. Higher magnification photo (right panel) shows immunoreactivity in the intercalated portion of acinus. **b** HAI-1 immunoreactivity in ADM. HE stain (left) and HAI-1 immunostain (right) are shown. **c** HAI-1 immunoreactivity in atypical ductal lesions. Bar, 50 μm in all photos

In normal pancreatic tissue, HAI-1 immunoreactivity was weak in the pancreatic duct epithelium and cells at intercalated portions of the acini (Fig. 1a; Additional file 1: Figure S1), and the immunoreactivity was mildly increased in the ductular epithelial cells in ADM near the tumor tissue (Fig. 1b). Immunoreactivity was observed on the apical (luminal) surface, basolateral surface or both. In atypical ductal lesions (pancreatic intraepithelial neoplasia: PanIN), HAI-1 immunoreactivity was enhanced compared to the adjacent non-dysplastic epithelium, and its basolateral localization was apparent (Fig. 1c).

**Fig. 2:**
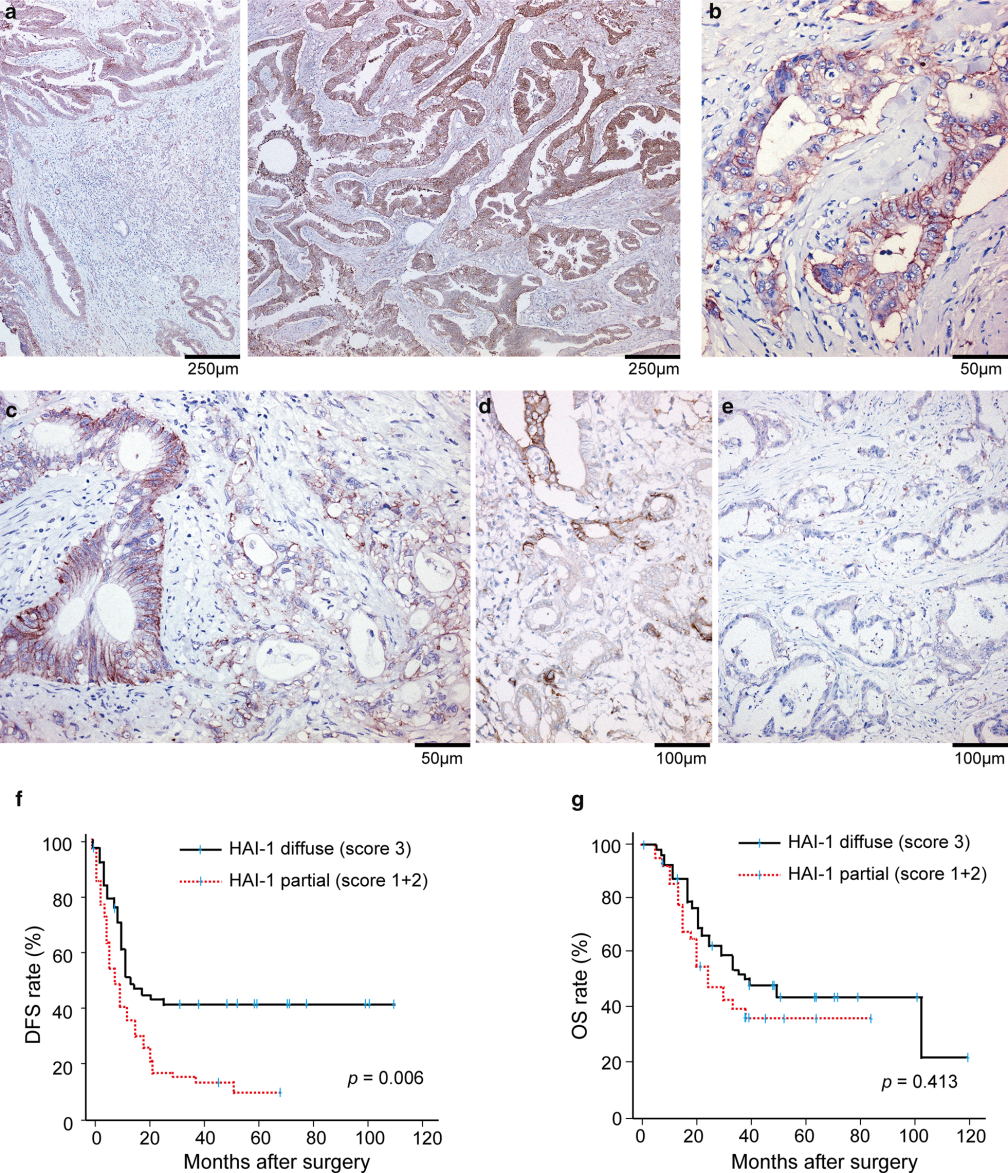
Immunoreactivity of HAI-1 in invasive PDAC cells. **a**, **b** Representative examples of diffuse immunoreactivity in cancer cells (score 3). Cell surface immunoreactivity of HAI-1 is evident. Bar, 250 μm (**a**) or 50 μm (**b**). **c**, **d** Reduction of HAI-1 immunoreactivity is present in some (**c**, score 2) or majority of (**d**, score 1) PDAC cells, showing intratumoral HAI-1 heterogeneity. Bar, 50 μm (**c**) or 100 μm (**d**). **e** Representative photo of HAI-1 negative case. Bar, 100 μm. **f**, **g** Association of diffuse HAI-1 immunoreactivity in PDAC cells to patient prognosis. Kaplan–Meier survival curves for DFS (**f**). Patients with diffusely positive HAI-1 staining (score 3, n = 39, total events 22) had favorable DFS compared with those with partial HAI-1 (score 1 + 2, n = 28, total events 25) (*p* = 0.006). Kaplan–Meier survival curves for OS (**g**). No significant difference was observed (*p* = 0.413). Total events are 22 and 28 for score 3 and score 1 + 2, respectively. *p* value was calculated by log-rank test. ^┼^censored cases

In PDAC cells, HAI-1 immunoreactivity tended to be stronger than normal duct epithelium and ADM. Among the 67 cases analyzed, 19, 9, and 39 cases had HAI-1 immunoreactivity scores of 1, 2, and 3, respectively. Therefore, diffuse HAI-1 expression (i.e., HAI-1 immunoreactivity score 3: Fig. 2a, b) was observed in 58% of PDAC cases. Whereas HAI-1 was frequently expressed in PDAC cells, reduced HAI-1 immunoreactivity was occasionally observed in cancer cells from the same PDAC case, indicative of intratumoral heterogeneity (Fig. 2c, d; Additional file 1: Figure S1). In three cases, HAI-1 immunoreactivity was hardly detectable in most cancer cells and only a faint immunoreactivity was observed focally (Fig. 2e).

We next analyzed the impact of HAI-1 immunoreactivity on clinicopathological parameters and patient prognosis. Diffuse HAI-1 expression (i.e., score 3) showed no correlation with major clinicopathological parameters, such as tumor size, the presence of lymph node metastasis, and tumor cell differentiation, and only older age (≥ 70) was correlated with the diffuse HAI-1 immunoreactivity (Table 1). Interestingly, PDAC cases with diffuse HAI-1 expression in the cancer cells showed favorable DFS compared with PDAC cases having partial or diffuse reduction of HAI-1 expression (score 1 + 2) (*p* = 0.006) (Fig. 2f). However, there was no significant difference regarding DFS between the cases of HAI-1 score 1 and HAI-1 score 2 + 3 (*p* = 0.106). On the other hand, OS was not altered by HAI-1 status (Fig. 2g). Univariate Cox proportional hazards regression analysis for the association between clinicopathological parameters (age, T factor, lymph node metastasis, tumor grade, adjuvant therapy, and HAI-1 status) and DFS showed significant impact of absence of lymph node metastasis (HR, 1.923; 95% CI 1.008–3.667; *p* = 0.047), administration of adjuvant therapy (HR, 4.452; 95% CI 2.432–8.151; *p* < 0.001), and diffuse HAI-1 status (HR, 0.429; 95% CI 0.241–0.765; *p* = 0.004) on favorable DFS. Then we carried out a multivariate analysis by incorporating parameters that showed a statistically significant effect in the univariate analysis. All three parameters showed significant effects on DFS, and diffuse HAI-1 immunoreactivity in PDAC cells was an independent prognostic factor for favorable DFS (HR, 0.375; 95% CI 0.208–0.676; *p* = 0.001) in this analysis.

**Table 1:** Association between clinicopathological parameters and diffuse HAI-1 immunoreactivity

Parameters	Cases	HAI-1 diffuse^a^	*p* value^b^
Gender			
Female	33	17	0.280
Male	34	22	
Age			
≥ 70	35	27	0.001
< 70	32	12	
T factor			
T1 + T2	11	7	0.690
T3 + T4	56	32	
Lymph node metastasis			
+	44	26	0.840
−	23	13	
Tumor grade			
Well/moderate	51	30	0.856
Poor	16	9	
Adjuvant therapy			
(+)	45	27	0.671
(−)	22	12	

^a^HAI-1 positive in more than 75% of cancer cells (score 3)

^b^Chi square test

### Discussion

This study analyzed HAI-1 expression in PDAC and its precursor lesion PanIN. The results revealed, for the first time, that HAI-1 expression is upregulated in PanIN lesions and is widely expressed in PDAC. On the other hand, around 40% of PDAC tumors had areas with reduced HAI-1 immunoreactivity, which predicted shorter DFS compared to the cases with diffuse (> 75% of cancer cells) HAI-1 expression. Although it is unclear why dysplastic intraductal lesions show increased cell surface HAI-1 immunoreactivity compared to normal duct epithelial cells, the enhanced HAI-1 expression may implicate enhanced expression of its cognate TTSPs in neoplastic cells. For example, matriptase is the best known cognate protease, and HAI-1 is paradoxically required for proper membrane localization and activation of matriptase acting as a molecular chaperone, even though HAI-1 inhibits matriptase after activation on the cell surface [3]. However, it is currently unknown which protease is indeed the target of HAI-1 in the pancreatic ductal epithelium, PanIN and PDAC.

PDAC usually has desmoplastic stroma, i.e., having many CAF, and thus, the biology of PDAC cells would be influenced by tumor cell-CAF interactions [4]. HGF is an example of a molecule involved in this kind of interaction, wherein cancer cells stimulate fibroblasts to produce HGF that in turn transduces signals through its specific receptor tyrosine kinase MET. However, because HGF is secreted as an inactive single-chain proform, extracellular proteolytic conversion to a two-chain active form is critical for its activity. HAI-1 inhibits major HGF-activating proteases such as HGFA, matriptase and hepsin that presumably act in tumor tissues [9]. Notably, HGFA-mediated HGF activation is upregulated in PDAC in response to hypoxic microenvironment [16], and matriptase expression is enhanced in PDAC tissue compared to the normal pancreatic tissue [17]. Therefore, insufficient HAI-1 levels can result in enhanced HGF-MET signaling in PDAC, which eventually promotes chemoresistance and invasive growth of the cancer cells [4, 9]. Moreover, matriptase also activates PDGF-D, macrophage stimulating protein (a ligand of RON receptor tyrosine kinase), protease-activated receptor 2 and urokinase-type plasminogen activator, all of which are likely involved in progression of PDAC [3, 14, 18], and matriptase-mediated activation of these molecules would be upregulated by HAI-1 insufficiency.

TMPRSS4 may be another candidate of the HAI-1 target in PDAC cells. Similar to matriptase and hepsin, TMPRSS4 belongs to a type II transmembrane serine protease family and was initially reported as a protein overexpressed in PDAC; this protein was originally termed TMPRSS3 [19]. Although the functions of TMPRSS4 is not well established, evidence is accumulating that it promotes cancer progression and EMT of cancer cells [3, 20]. Indeed, we previously reported that HAI-1 deficiency-induced EMT of a PDAC cell line (SUIT2) was abrogated by *TMPRSS4* gene silencing [12].

The mechanism by which cell surface HAI-1 levels are decreased in some PDAC cells remains to be determined. Two mechanisms have been implicated for the decreased membranous HAI-1 in cancer cells: (1) reduced transcription and (2) enhanced shedding. For transcription reduction, the existence of a transcription repressor in the *SPINT1* gene and hypermethylation of the *SPINT1* promoter have been reported. For example, a TMPRSS2/ERG fusion gene product suppressed *SPINT1* gene transcription in prostate cancer [21]. Lung cancer cells with an EMT phenotype show hypermethylation of the *SPINT1* promoter [10]. The shedding of cell surface HAI-1 is mediated by a metalloprotease [8], and membrane type-1 matrix metalloprotease (MT1-MMP) is a likely candidate for HAI-1 sheddase in cancer cells [11, 22]. In fact, the MT1-MMP expression is upregulated in PDAC cells and is involved in invasiveness and desmoplastic reaction [23]. Therefore, those regions of cancer that show reduced cell surface HAI-1 may have enhanced MT1-MMP activity.

### Conclusions

This study revealed that HAI-1 is upregulated in PDAC precursor lesions compared with normal duct epithelium, and is broadly expressed in PDAC. On the other hand, diffuse immunoreactivity of cell surface HAI-1 in PDAC may predict favorable DFS.

### Limitations

Our study included a limited number of patients (67 cases), and there is no formal sample size determination. Therefore, we could not perform disease stage-controlled rigorous statistical analysis regarding the impact of HAI-1 immunoreactivity to patients’ prognosis.

## Additional file

**Additional file 1: Figurre S1.** Specificity of HAI-1 antibody. To confirm the specificity of HAI-1 immunoreactivity, representative negative control (non-specific IgG) photos of weakly HAI-1-positive cases are shown. **A**, Non-neoplstic duct epithelium. **B**, PDAC cells with weak HAI-1 immunoreactivity.

## Abbreviations

HAI-1: hepatocyte growth factor activator inhibitor type 1
PDAC: pancreatic ductal adenocarcinoma
DFS: disease-free survival
CAF: cancer-associated fibroblasts
HGF: hepatocyte growth factor
EMT: epithelial to mesenchymal transition
HGFA: HGF activator
OS: overall survival
ADM: acinar–ductal metaplasia
HRs: hazard ratios
Cis: confidence intervals
PanIN: pancreatic intraepithelial neoplasia
