# Supplementary material for: Prognostic significance of hepatocyte growth factor activator inhibitor type 1 (HAI-1) immunoreactivity in pancreatic ductal adenocarcinoma
Source: BMC Res Notes. 2017 Dec 4;10:674. doi: 10.1186/s13104-017-3014-x (PMC5715503; doi:10.1186/s13104-017-3014-x)
Supplement: Supplementary file 1 — Additional file 1: Figurre S1. Specificity of HAI-1 antibody. To confirm the specificity of HAI-1 immunoreactivity, representative negative control (non-specific IgG) photos of weakly HAI-1-positive cases are shown. A, Non-neoplstic duct epithelium. B, PDAC cells with weak HAI-1 immunoreactivity. [file 13104_2017_3014_MOESM1_ESM.pdf]

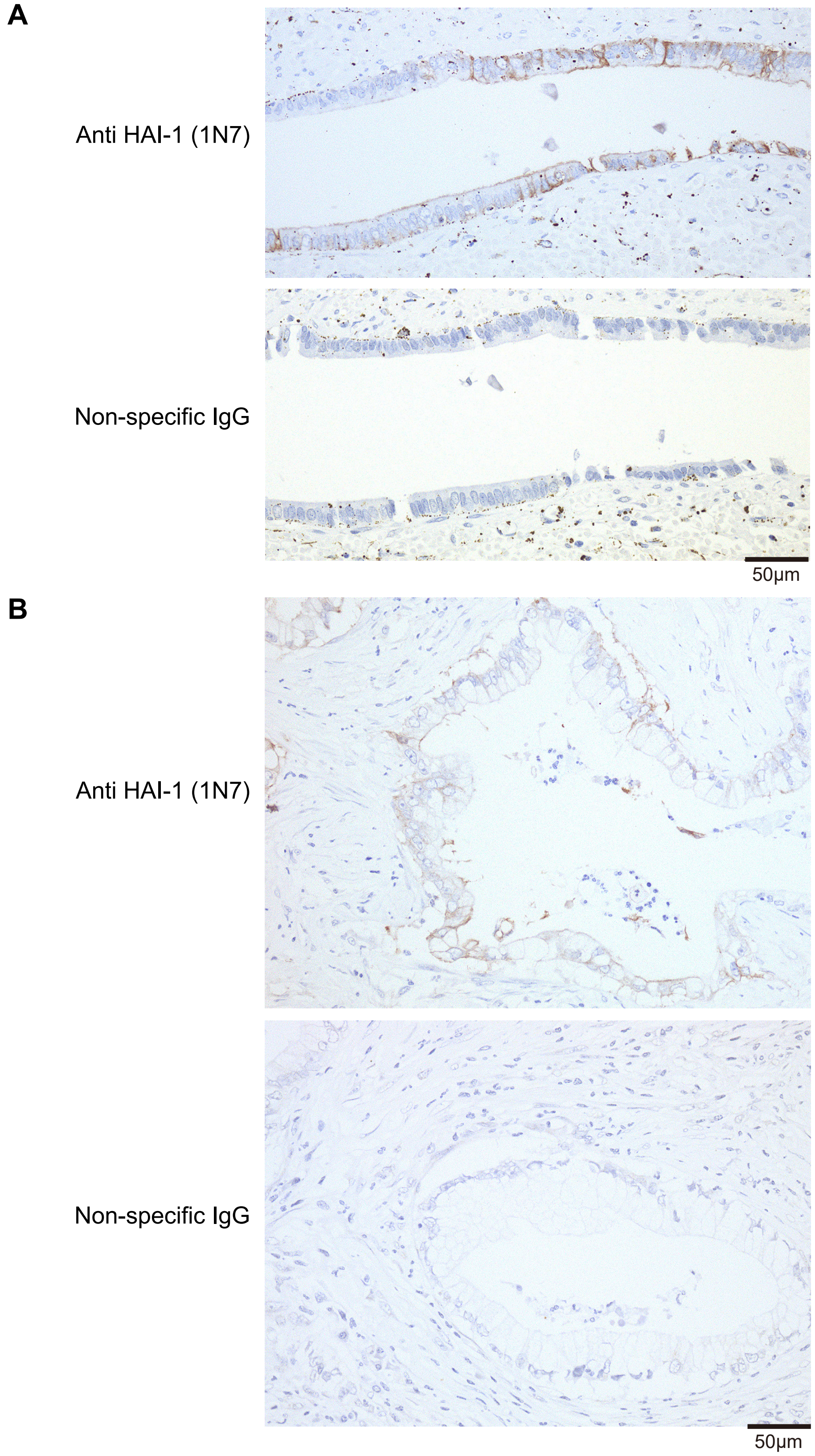

Supplementary Figure S1.
Specificity of HAI-1 antibody. To confirm the specificity of HAI-1 immunoreactivity, representative negative control (non-specific IgG) photos of weakly HAI-1-positive cases are shown. **A**, Non-neoplstic duct epithelium. **B**, PDAC cells with weak HAI-1 immunoreactivity.
